# Restriction of AID activity and somatic hypermutation by PARP-1

**DOI:** 10.1093/nar/gkz466

**Published:** 2019-05-25

**Authors:** Sandra Tepper, Oliver Mortusewicz, Ewelina Członka, Amanda Bello, Angelika Schmidt, Julia Jeschke, Arthur Fischbach, Ines Pfeil, Svend K Petersen-Mahrt, Aswin Mangerich, Thomas Helleday, Heinrich Leonhardt, Berit Jungnickel

**Affiliations:** 1 Department of Cell Biology, Institute of Biochemistry and Biophysics, School of Biology and Pharmacy, Friedrich Schiller University, 07745 Jena, Germany; 2 Department of Biology II and Center for Integrated Protein Science Munich (CIPSM), Ludwig Maximilians University Munich, 82152 Planegg-Martinsried, Germany; 3 Science for Life Laboratory, Department of Oncology-Pathology, Karolinska Institutet, S-171 76 Stockholm, Sweden; 4 Department of Biology, University of Konstanz, 78457 Konstanz, Germany; 5 Institute of Clinical Molecular Biology, Helmholtz Center Munich, German Research Center for Environmental Health, 81377 Munich, Germany; 6 DNA Editing in Immunity and Epigenetics, IFOM-Fondazione Instituto FIRC di Oncologia Molecolare, Milano, Italy

## Abstract

Affinity maturation of the humoral immune response depends on somatic hypermutation (SHM) of immunoglobulin (Ig) genes, which is initiated by targeted lesion introduction by activation-induced deaminase (AID), followed by error-prone DNA repair. Stringent regulation of this process is essential to prevent genetic instability, but no negative feedback control has been identified to date. Here we show that poly(ADP-ribose) polymerase-1 (PARP-1) is a key factor restricting AID activity during somatic hypermutation. Poly(ADP-ribose) (PAR) chains formed at DNA breaks trigger AID-PAR association, thus preventing excessive DNA damage induction at sites of AID action. Accordingly, AID activity and somatic hypermutation at the Ig variable region is decreased by PARP-1 activity. In addition, PARP-1 regulates DNA lesion processing by affecting strand biased A:T mutagenesis. Our study establishes a novel function of the ancestral genome maintenance factor PARP-1 as a critical local feedback regulator of both AID activity and DNA repair during Ig gene diversification.

## INTRODUCTION

Genome maintenance is essential for the prevention of cancer and early aging (1,2). To deal with the multitude of endogenous and exogenous threats to genome integrity, a limited set of pathways with the capacity to repair defined lesions has evolved, which is regulated by lesion type, cell cycle phase and checkpoint signaling. Poly(ADP-ribose) polymerase (PARP) proteins are evolutionarily old genome maintenance factors contributing to some of these repair pathways and their control, among other functions in transcription, epigenetics and immune homeostasis (3,4). PARPs bind to single strand breaks in the DNA, where they catalyze the transfer of ADP-ribose units from NAD^+^ to themselves and other acceptor proteins, forming long branched poly(ADP-ribose) polymers (PAR) that lead to the local recruitment and control of PAR-binding repair factors (5). PARP-1, the founding member of a family of presently 18 PARPs, is responsible for ≈90% of PAR synthesis upon DNA damage (6) and is thus a key DNA repair and genome maintenance factor.

In the adaptive immune system of vertebrates, targeted genetic changes of intricate complexity allow for the formation of antigen receptors capable of detecting and eliminating virtually all pathogens (7,8). V(D)J recombination in B and T cell precursors in primary lymphoid organs combines a modular architecture of antigen receptor gene loci with the capacity of a hijacked transposase (Rag1/2) and highly erroneous non-homologous end joining (NHEJ) to effect gene recombination for the generation of a multitude of antigen receptors (9). Ig gene conversion occurring in some farm animals such as chickens may modify the resultant V(D)J joint of Ig genes via rather promiscuous homologous recombination that leads to the integration of segments from variant upstream pseudogenes into the V(D)J region (10). Class switch recombination (CSR), which occurs upon acute infections to change antibody effector functions, is once again based on deletion-focused NHEJ (11).

The most striking example of erroneous DNA repair in adaptive immunity is somatic hypermutation (SHM), the basis of affinity maturation of humoral immunity. Here, activation-induced deaminase (AID) (which also initiates Ig gene conversion and CSR) triggers cytosine deamination to form uracils in transcribed Ig loci (12). These uracils are the basis for three distinct processing pathways leading to different mutational outcomes (13): (i) replication over the uracils leads to transition mutations at C:G residues (termed phase 1A of SHM); (ii) removal of the uracil by uracil-DNA glycosylase (UNG), followed by translesion synthesis over the abasic site, allows for C:G transversions in addition (phase 1B); (iii) processing of the AID-mediated U:G mismatch via non-canonical mismatch repair (14) mainly involving the translesion polymerase Polη leads to mutations at A:T residues (phase 2). Overall, this system allows for a mutation rate roughly 10^6^ times higher than spontaneous mutagenesis in vertebrate genomes. Stringent selection of B cells with high affinity receptors eventually leads to affinity maturation of the humoral adaptive immune response (15).

While the molecular mechanisms triggering error-prone instead of error-free repair during SHM are largely elusive to date, mechanisms regulating AID activity are extensively studied and involve expression regulation via various transcription factors and miRNAs, balancing of cellular localization by cytosolic retention and nuclear import factors, as well as regulation of AID’s nuclear stability and its targeting to Ig genes (16–19). We have recently shown that PARP-1 is involved in AID regulation upon exogenous DNA damage, effectively leading to sequestration and stabilization of this mostly cytoplasmic enzyme in the cell nucleus (20). In the present study, we have investigated whether PARP-1 also affects AID regulation in the physiological context of Ig diversification. We show that PARP-1 is indeed a restriction factor of AID activity at the Ig locus, mediating its PARylation-dependent trapping at DNA damage sites via AID-PAR association and thus limiting further AID induced damage induction at its site of action. Upon PARP-1 inactivation, B cells show higher AID activity at the Ig locus, concomitant with increased overall SHM and a pattern shift indicating a loss of strand bias of the A:T mutator. Our findings identify a novel key regulation mechanism of AID during SHM and shed light on a previously unanticipated local pathway of genome maintenance in hypermutating cells.

## MATERIALS AND METHODS

### Co-immunoprecipitation

Raji (ATCC^®^ CCL-86™) and BJAB (obtained from the Helmholtz Center Munich) cells were cultured at 37°C in RPMI 1640 medium supplemented with 10% fetal calf serum (FCS, Sigma), 100 U/ml penicillin/100 μg/ml streptomycin (Invitrogen), 2 mM glutamine (Invitrogen) and 1 mM sodium pyruvate (Invitrogen) at a cell density of 2–5 × 10^5^/ml. Where indicated, 0.1% of MMS (Merck) was added 1 h before processing. Cells were then sedimented for 5 min, washed twice in PBS and resuspended in lysis buffer (30 mM Tris–HCl, pH 7.5; 150 mM NaCl; 10% glycerol; 10 μM ZnCl_2_; 1.5 mM MgCl_2_; 1 μM β-mercaptoethanol; 0.5% IGEPAL or NP40, 15 U/ml DNase, protease and phosphatase inhibitor cocktail) and incubated for 60 min at 4°C on a stirring wheel, followed by centrifugation (10 min at 15 000 rpm) and separation of the supernatant. Protein amounts were adjusted to 3500 μg for all samples using the Bio-Rad DCTM Protein Assay-Kit. 50 μl of ProteinG-Sepharose beads (VWR) were washed with lysis buffer and 2.8 μl affinity-purified α-AID antibody (clone EK2/2H5) was added in 500 μl of lysis buffer. The beads were incubated for >1 h on a stirring wheel at 4°C. After centrifugation, cell lysates were incubated with the beads overnight on a stirring wheel at 4°C. Beads were washed 12 times with 500 μl of lysis buffer (like above but with 300 mM NaCl) before boiling in 2× SDS-PAGE sample buffer (150 mM Tris–HCl pH 6,8, 1,2% SDS, 30% glycerin and 0.1 M DTT). Western blot analyses used the 5G9 antibody for AID (clone EK2/5G9) (21) and a α-PARP-1 (E78) (ab32071, Abcam) antibody.

### PAR overlay assay

PAR overlay assays were performed as described previously (22). The indicated amounts of commercial histone H1 (Abcam, ab198676 or Sigma, H1917) and BSA (Roth, 8076) proteins or of purified recombinant AID protein (23,24) that was boiled in 2× SDS buffer (150 mM Tris–HCl pH 6.8, 1.2% SDS, 30% glycerin and 0.1 M DTT), were slot-blotted onto a nitrocellulose membrane (Roth, 0031). The membrane was incubated overnight at 4°C in TBST buffer (150 mM NaCl, 10 mM Tris pH 8, 0.05% Tween 20) containing 0.2 μM PAR (synthesized and purified as described previously (25)). The membrane was washed three times for 10 min in TBST high salt buffer (TBST containing 1 M NaCl), twice for 10 min in TBST, and subsequently blocked for 1 h at RT in TBST supplemented with 5% milk powder. PAR was detected using the monoclonal 10H anti-PAR antibody (Enzo, ALX-804-220).

### Live cell imaging

Live cell microscopy and microirradiation experiments in HeLaKyoto and U2OS cells were performed as previously described (26,27). In short, either an UltraVIEW VoX spinning disc microcope (Perkin Elmer) with a Plan-Apochromat 63×/1.4 Oil objective or a Zeiss LSM780 confocal laser scanning microscope, equipped with a Plan-Apochromat 40×/1.30 Oil DIC M27 objective were used. Both microscopes were equipped with a heated environmental chamber set to 37°C. Cells were seeded into μ-Grid (35 mm with Grid, ibidi) dishes and pre-sensitized before laser microirradiation with 10 μg/ml Hoechst for 10 min. DNA damage in a 10 pixel diameter spot within the nucleus was induced with a 405 nm diode laser set to 77% (Spinning disk microscope) or 100% (LSM780 confocal microscope). Transfection with the HA-AIDΔNES-GFP plasmid and the R19E/R24E version of this plasmid (generated as described in ref. 20) was done 24–48 h before the microirradiation experiment. For knockdown experiments, cells were transfected with control siRNA (Qiagen, SI03650325, AATTCTCCGAACGTGTCACGT) or PARP-1 siRNA (PARP-1_5, Qiagen, SI02662989, ACGGTGATCGGTAGCAACAAA) 72 h before performing laser microirradiation experiments at a concentration of 10 nM using Interferrin (PolyPlus). PARP-1 inhibitors 3-AB (1 mM) and Olaparib (10 μM) were added 1 or 3 h prior to laser microirradiation, whereas LMB (20 ng/ml) was added 6 h before laser microirradiation. Data of at least 13 nuclei from two independent experiments were averaged and the mean curve and the standard error of the mean calculated and displayed.

### F3H assay

To anchor GFP and AID-ΔNES-GFP to a defined nuclear structure, we employed the previously described F3H assay (28). U2OS 2-6-3 (29) cells were co-transfected with GBP-LacI and with either GFP or AID-ΔNES-GFP expression vectors. After 24 h, 1 μM Olaparib, 1 mM 3-AB or equal amounts of DMSO was added to the cells. 2-6-3 cells were fixed in 4% PFA after 24 h, permeabilized in 0.5% Triton X-100 and probed with antibodies for γH2AX (Millipore, 05-636) and 53BP1 (Abcam, ab36823) for 1 h at RT. After three washes in PBS, secondary antibodies donkey-anti-rabbit Alexa Fluor 568 (Life Technologies, A10042) and donkey-anti-mouse Alexa Fluor 647 (Life Technologies, A31571) were added for 1 h at RT together with DAPI. Cells were imaged on a ZEISS LSM780 with a Plan-Apochromat 40×/1.3 Oil DIC M27 objective and sequential scanning. Co-localization of GFP, γH2AX and 53BP1 was determined using line scans and fluorescence intensities at the LacI focus measured using ImageJ. Data of three independent experiments were analysed using Microsoft Excel and displayed with GraphPad Prism. Statistical significance was determined using Student's *t*-test.

### Measurement of AID activity and Ig diversification in DT40 cells

DT40Cre1 cells and DT40ψV^−^ cells obtained from H. Arakawa (30), and DT40UNG^−/−^ cells obtained from H. Saribasak (31) were cultured at 41°C in RPMI 1640 medium supplemented with 10% FCS, 1% chicken serum, 0.1 mM β-mercaptoethanol, 100 U/ml penicillin/100 μg/ml streptomycin, 2 mM glutamine and 1 mM sodium pyruvate at a cell density of 2–5 × 10^5^/ml. For analysis of AID and Ig diversification activity, the cells were seeded at very low density into 96 well plates to obtain single cell clones. Where indicated, 0.1% DMSO without additive or containing 2.5 or 5 μM TiqA (Santa Cruz), 25 or 50 μM NU1025 (Santa Cruz) were included in all culture media. Single cell clones were transferred to 24 or 48 well plates after 7–10 days and fed every 3–4 days. FACS analyses were performed after staining with α-chicken IgM-PE (Southern Biotech, SBA-8310-09), and data were analyzed with FlowJo software. FACS data were excluded if clones exceeded 85% dead cells or came below 80% of AID-GFP positivity. Representative subclones (close to the mean) were used for sequence analyses of AID activity (DT40UNG^−/−^) after 20 days of culture. Genomic DNA was isolated (QIAamp DNA Mini Kit) and the rearranged light chain λ locus was amplified with Phusion High-Fidelity DNA Polymerase (Thermo Scientific) and the following primers: 5′-TGG GAA ATA CTG GTG ATA GGT GGA T-3′ and 5′-CCT CCAT TTT TTG ACA GCA CTT ACC TGG ACA GCT G-3′. PCR Products were cloned into the pGEM^®^-T vector (Promega) and sequenced with the primer 5′-GAG CGC AGG GAG TTA TTT GCA TAG-3′. Geneious software was used for sequence alignments and identification of mutations. Data were not used if sequences were of insufficient quality or too short.

### Inactivation of PARP-1 in DT40UNG^−/−^ cells

The 5′ arm for the targeting vector was amplified with the primers #1 5′-CCG CTC GAG AGG ACT CGC TGC GCC TGG CCC T-3′ and #2 5′-CGC GGA TCC TCA GTG AGG GAC TTT GCC ATC GAA C-3′, while the 3′ arm was amplified with the primers #3 5′-TGG GGA TTG TCT GCC AAG AAG-3′ and #4 5′-CAG CCG TTA AAA TGG CTC AGA TT-3′. Arms were restricted by XhoI and BamHI or SpeI and BamHI, cloned into the pBluescriptKS vector (Stratagene), and the resultant BamHI site between them served to insert loxP-flanked resistance cassettes (from pLoxBsr, pLoxGpt) (32). After restriction with NotI, plasmids were transfected into DT40 cells with a BioRad gene pulser set at 50 μF and 800 V. Selective media containing 5 μg/ml blasticidin S HCl (MoBiTec GmbH) or 30 μg/ml mycophenolic acid (gpt; VWR) were added 1 day after transfection, and transfected single cell clones were isolated and further cultured for 10–14 days. For PCR confirmation of targeted integration, cells from 0.5 ml of culture were pelleted and genomic DNA was isolated with the QIAamp DNA Mini Kit (QIAGEN). PCRs were performed with the following primer combinations: #R^BSR^ 5′-ACT GCA TTC TAG TTG TGG TTT GTC C-3′ or #R^GPT^ 5′-CGC CGG ACG AAC TAA ACC TGA C-3′ and #4 for checking directed integration of the resistance cassette; #3 and #4 for amplification of the endogenous locus (3′ arm) and #5 5′-GGA AGG AGG TTG GCA AGG CT-3′ and #6 5′-CTG TGT GGC CCC ATA TGC T-3′ to amplify endogenous exons that should be replaced by the resistance cassette if the targeting construct is fully integrated via homologous recombination. Western blot analyses were performed with α-PARP-1 (E87) (ab32071, Abcam), and α-actin (A-2066, Sigma-Aldrich) and α-AID (clone EK2/5G9) antibodies.

### Analysis of SHM in RAMOS cells

RAMOS cells (obtained from M. Neuberger, Cambridge) were cultured at 37°C in RPMI 1640 medium supplemented with 10% FCS, 100 U/ml penicillin/100 μg/ml streptomycin, 2 mM glutamine and 1 mM sodium pyruvate (all Invitrogen) at a cell density of 2–5 × 10^5^/ml. For hypermutation assays, the cells were seeded at low density into 96-well plates to obtain single cell clones and constantly supplemented with 0.1% DMSO without additive or containing 5 or 10 μM TiqA (Santa Cruz), 25 μM or 50 μM NU1025 (Santa Cruz). Cells were transferred to 48-well plates after 10–14 days, and fed every 3–4 days. FACS analyses were performed using anti human IgM-FITC antibodies (Sigma, F5384). DNA of representative single cell clones was isolated after 42 days, and the rearranged V_H_DJ_H_ region was amplified with the primers 5′-CAG GGT ACC CCC AAG GTG AGC CCA AAA GA-3′ and 5′-CGG GAT CCC GCA TCG GGG CCG ACA GCA CT-3′, cloned into the pGEM^®^-T vector (Promega), sequenced with the primer 5′-CTC CTG GTG GCA GCT CCC AGA T-3′ and analyzed with Geneious software. SHM frequencies were acquired and calculated manually.

### Analysis of class switching in CH12F3 and primary mouse cells

CH12F3 cells were cultured at 37°C in RPMI 1640 medium supplemented with 10% FCS, 100 U/ml penicillin/100 μg/ml streptomycin (Invitrogen), 0.05 mM β-mercaptoethanol (Sigma) and 10 mM HEPES (ThermoFisher or Invitrogen). CH12F3 cells were stained with a 1:10000 dilution of CFSE (Thermo Scientific) and incubated with 0.1 μg/ml αCD40 (eBiosciences), 5 ng/ml IL-4 (eBiosciences) and 10 ng/ml TGF-β1 (NEB) for 2 days in medium supplemented with 0.1% DMSO without additive or containing 5 μM or 10 μM TiqA, 25 μM or 50 μM NU1025, followed by FACS analysis using α-mouse IgA-PE antibody (eBioscience, 12-4204-81). Primary mouse B cells (WT and PARP-1^−/−^) were isolated using MACS depletion with anti-CD43 microbeads (Miltenyi Biotec), cultured in CH12F3 medium with 1 μg/ml αCD40 and 20 ng/ml IL-4 in order to induce class switching to IgG1 within 3–4 days, partially under the influence of DMSO and PARP inhibitor additions as above. FACS analyses for CSR were performed after staining with α-mouse sIgG1-FITC antibody (BD Pharmingen, 553443). Supernatants of WT and PARP-1^−/−^ cells were used for quantification of the secreted IgG1/IgM antibodies by ELISA. Plates were coated with α-mouse IgG1/IgM antibody (BD Pharmingen, 553445/553435) prepared in carbonate buffer (pH 9.5; 0.1 M Na_2_CO_3_; 0.1 M NaHCO_3_). Detection included incubation with anti-IgG1/IgM-biotin (BD Pharmingen, 553441/553406) (30 min) followed by HRP Streptavidin (BioLegend) (1 h) and an *O*-phenylenediamine substrate solution (Sigma) (30 min in the dark), was stopped with 3 N HCl and measured at 492 nm. Analyses were done in duplicates with three different sample dilutions each.

### Analysis of SHM in mice

WT and PARP-1^−/−^ mice on a 129S2/SvPas background were obtained from Jackson labs and bred in an SPF facility. All animal experiments were approved by the appropriate institutional and governmental committees for animal welfare (Thüringer Landesamt für Verbraucherschutz). Immunizations were performed i.p. to male and female mice at the age of 8–12 weeks with alum-precipitated nitrophenylacetyl chicken gamma globulin (NP-CGG ratio > 40; Biocat), and successful immunization was checked in blood samples at day 7 and 14 by an NP-specific (NP3 or NP15 (Biocat)) ELISA. B cells were isolated from the spleen by MACS depletion with α-CD43 microbeads (Miltenyi Biotec) and stained with α-CD95-PE, α-B220-PerCP (both BD Pharmingen, 554258 and 553093) and α-PNA-FITC (Vector Laboratories) before sorting with a FACSAria. The JH4 intron was amplified using #V186.2 fdw 5′-CAG TAG CAG GCT TGA GGT CTG GAC-3′ and #JH_4_-rev 5′-CTC CAC CAG ACC TCT CTA GAC AGC-3′ or a nested PCR strategy with the primers #JH_4_-fwd 5′-CAG CCT GAC ATC TGA GGA CTC TGC-3′ and #JH_4_-rev for the first round, whereas the second round was performed with #JH_4_-fwd2 5′-ACT ACT GGG GTC AAG GAA CCT CAG-3′ and #JH_4_-rev. Gel-purified products were cloned into the pGEM®-T vector (Promega) and individual colonies were picked and sequenced using the #JH_4_-rev primer. Hypermutation frequencies and pattern analyses based on unique mutations were acquired manually with Geneious software (Biomatters) and confirmed with the SHM Tool (33) (http://shmtool.montefiore.org/cgi-bin/p1). Whenever clonally identical mutated sequences were found, all but one were excluded from the analysis to rule out the influence of highly abundant B cell clones on the mutational pattern. Data were also not used if sequences were of insufficient quality or too short.

## RESULTS

### PARP-1 mediated PARylation recruits AID to DNA damage sites

**Figure 1.**
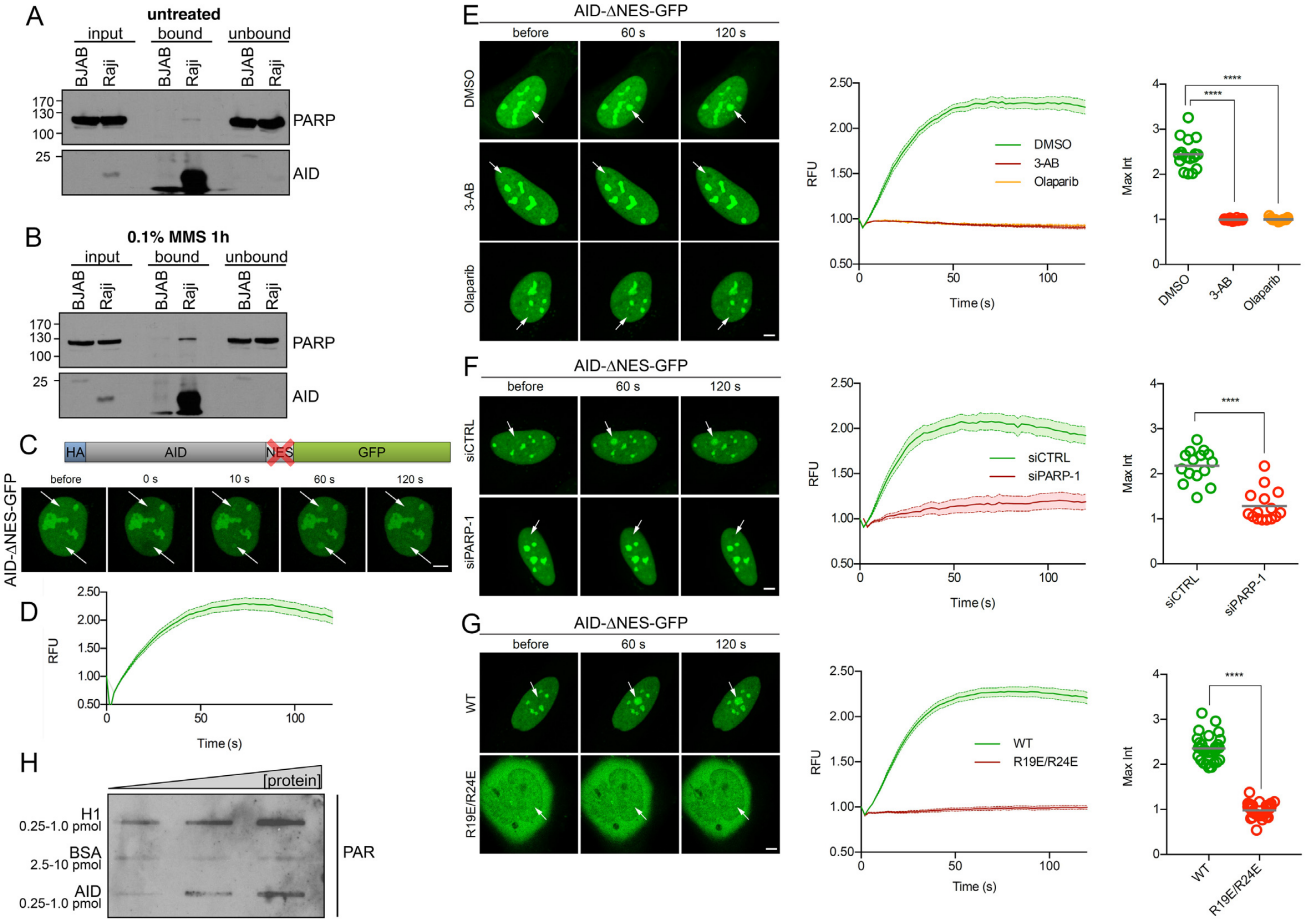
PARP-1 activity recruits AID to sites of DNA damage. (**A**) Co-immunoprecipiation of AID and PARP-1 in AID-expressing Raji cells and AID^low^ BJAB cells in the absence of DNA damage, using an antibody directed against the AID C-terminus. Western blot detection of PARP-1 and AID is shown. Data are representative of two independent experiments. (**B**) Co-Immunoprecipiation as shown in A), after treating the cells with 0.1% MMS for 1 hour. The experiment is representative of three independent experiments and was performed in parallel to the one shown in A). (**C**) Representative pictures of live cell imaging experiments showing recruitment of AID to sites of laser microirradiation (arrows) in HelaKyoto cells transiently transfected with a nuclear-restricted AIDΔNES-GFP construct. (**D**) Quantification of the results shown in (C). (**E**) Inhibition of AIDΔNES-GFP recruitment to DNA damage sites in U2OS cells by the PARP inhibitors Olaparib and 3-AB. F) Inhibition of AIDΔNES-GFP recruitment to DNA damage sites in U2OS cells by a siRNA to PARP-1. (**G**) Mutation of arginine residues 19 and 24 in AID abolishes AIDΔNES-GFP recruitment to DNA damage sites in U2OS cells. H) PAR overlay assay after slot-blotting of purified proteins at increasing amounts. Bound purified PAR chains are detected with an antibody. H1 and BSA serve as positive and negative controls, respectively. Data are representative of two independent experimental replicates. For microirradiation experiments, data of at least 13 cells from two independent experiments are shown as mean ± SEM. Microirradiation was performed using a Spinning disk (C, D) or confocal microscope (E–G). Statistical significance was determined using Student's *t*-test (****P< 0.0001). Scale bar, 5 μm. RFU = relative fluorescence units. Max Int = maximum intensity.

As we previously found that PARP-1 regulates AID localization and stability upon exogenous DNA damage (20), we first asked whether the two enzymes interact in B cells undergoing SHM. Co-immunoprecipitation studies revealed that this is indeed the case: In the hypermutating and AID-expressing cell line Raji (34), immunoprecipitation with an AID antibody co-precipitated PARP-1, while this was not the case in non-mutating and AID^low^ BJAB cells (Figure 1A). Interestingly, the interaction of PARP-1 and AID was more pronounced when the cells were treated with methyl methanesulfonate (MMS), implying enhanced AID-PARP-1 association upon DNA damage (Figure 1B).

PARP-1 exerts its functions to a large extend by substantial autoPARylation (35), leading to PAR-binding-dependent recruitment of DNA repair factors to sites of DNA damage identified by PARP-1. While AID is certainly not a classical DNA repair factor, we wondered whether a similar mechanism might lead to its recruitment to DNA damage sites. In HelaKyoto cells carrying a nuclear AID-ΔNES-GFP fusion lacking the C-terminal nuclear export sequence, laser microirradiation indeed led to an accumulation of the AID fusion protein at induced DNA damage foci (Figure 1C and D). A similar recruitment could be observed in U2OS cells (Figure 1E). Strikingly, this effect was blocked completely when the PARylation activity of PARP was inhibited using the nicotinamide mimetics 3-aminobenzamide (3-AB) or Olaparib (Figure 1E), (36,37), and AID even appeared to dissociate from damaged sites in this situation. Also, AID accumulation at damage sites did not occur to any significant extent in cells with siRNA-mediated PARP-1 depletion (Figure 1F). Interestingly, full length GFP-tagged AID translocated from the cytoplasm to the nucleus upon laser microirradiation (Supplementary Figure S1A and B), and inhibition of its nuclear export by leptomycine B (LMB) also resulted in accumulation of AID-GFP at laser-induced DNA damage sites (Supplementary Figure S1C and D) with kinetics similar to PARP-1 itself (38). We thus conclude that AID can be recruited to DNA lesions by PARP-1-mediated PARylation, reminiscent of the recruitment of DNA repair factors.

Recruitment of repair proteins via PARylation occurs based on their interaction with PAR chains, resembling protein/DNA interactions. AID may in principle bind to PAR chains directly or via another protein. AID contains a substrate channel involved in AID-DNA binding and an assistant patch for binding of structured DNA during class switch recombination (39). Strikingly, mutagenesis of the two most highly conserved arginines (R19/R24) in the AID substrate channel abolished recruitment of AID to laser-induced DNA damage sites (Figure 1G). This finding suggests that direct AID-PAR interaction via the substrate channel may be involved in AID recruitment to DNA damage sites. Indeed, PAR overlay assays revealed that purified AID protein may directly associate with purified PAR chains (Figure 1H), providing a very direct mechanism for AID recruitment to DNA damage sites by PARP-1.

### PARP limits AID-mediated DNA damage induction at its site of action

**Figure 2.**
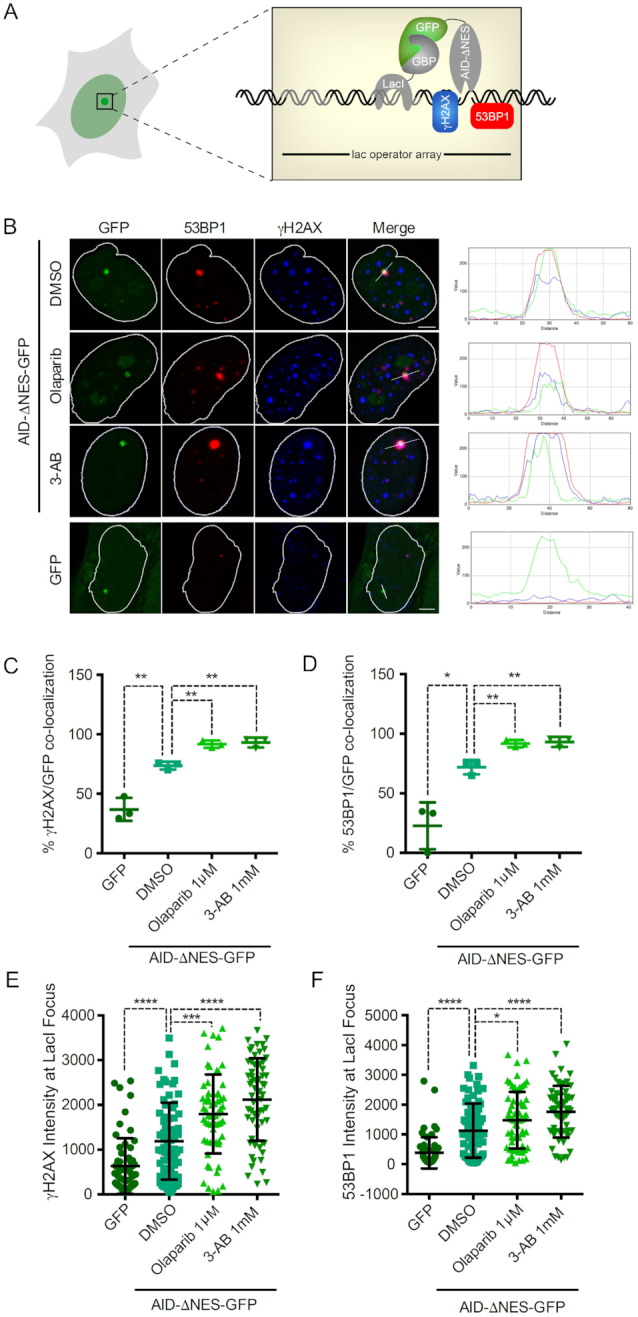
PARP limits local AID mediated DNA damage induction. (**A**) Schematic of the F3H experimental system used to localize AID-ΔNES-GFP to a defined nuclear structure. GFP fusion proteins are tethered to a lac array in U2OS 2-6-3 cells by a GFP-binding protein-LacI fusion, and the DNA damage caused is detected by γH2AX or 53BP1 staining. (**B**) Representative confocal images of U2OS 2-6-3 cells expressing either GFP alone or AID-ΔNES-GFP treated with DMSO, 1 μM Olaparib or 1 mM 3-AB for 24 h. DNA damage induction via AID was visualized using antibodies against γH2AX and 53BP1. (C and D) Percentage of cells displaying co-localization of AID-ΔNES-GFP or GFP with γH2AX (**C**) and 53BP1 (**D**), respectively. Data from three independent experiments are shown. (E and F) Scatter plot of fluorescence intensities of γH2AX (**E**) and 53BP1 (**F**) at the Lac operator array in 2-6-3 cells expressing AID-ΔNES-GFP or GFP. Data from >60 cells gathered in three independent experiments are shown. Statistical significance was determined using the Student's *t*-test (*P< 0.05; **P< 0.01; ***P< 0.001; ****P< 0.0001). Error bars indicate the standard deviation.

Recruitment of a potent mutator to sites of DNA damage might serve to either boost its function, or else to limit its activity until repair is achieved. Involvement of the DNA binding substrate channel in AID-PAR interaction might suggest blockade of AID function upon PAR-mediated recruitment, as only one polymer may be bound at a time. To formally assess which of the two scenarios mentioned above holds true for the effect of PARP on AID function, we used the previously described F3H assay (28). A high-affinity GFP-binding nanobody (GBP) is coupled to the Lac repressor (LacI). In 2-6-3 cells, which harbour a stably integrated lac operator array (*LacO*), the GBP-LacI protein binds to the *LacO* and recruits any GFP-fusion protein that is co-expressed to this *LacO* region in the nucleus (Figure 2A). Co-expression of AID-ΔNES-GFP with GBP-LacI in 2-6-3 cells resulted in tethering of the AID fusion to *LacO*, which is visible as a distinct green spot in the nucleus (Figure 2B). Interestingly, local accumulation of AID-ΔNES-GFP led to induction of DNA damage visualized by γH2AX and 53BP1 co-localization with AID at the *LacO* (Figure 2B–F). Importantly, inhibition of PARP-1 catalytic activity with two different inhibitors (Olaparib and 3-AB) led to a further significant increase in DNA damage induction compared to the DMSO control (Figure 2C–F). This finding indicates that PARP-1 may locally restrict AID activity, limiting the amount of DNA damage induced by AID.

### PARP-1 activity restricts AID activity at the Ig locus

**Figure 3.**
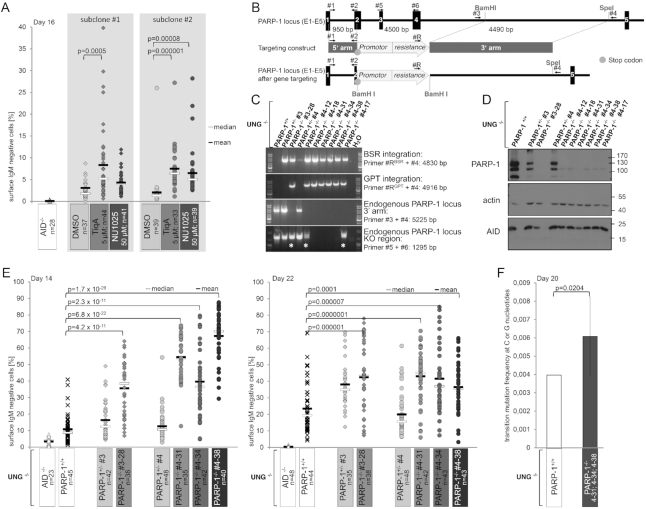
PARP-1 activity inhibits AID activity at the Ig locus. (**A**) Effects of PARP inhibition on AID activity in DT40UNG^−/−^ cells. Each dot indicates %sIgM loss in one single cell clone (see Supplementary Figure S2A for experimental system). Data represent three independent experiments. Statistically significant differences are marked with brackets, and P-values are derived from a two-sided Student's *t*-test. (**B**) Strategy for inactivation of PARP-1 in DT40UNG^−/−^ cells. Exons are marked as boxes, primers used for constructing the knockout vector and for genotyping of targeted clones are indicated. (**C**) Detection of WT and targeted alleles in parental, heterozygous and homozygous cells by the PCR approaches depicted in B). Asterisks mark clones in which one arm of the targeting vector did not integrate by homologous recombination. (**D**) Western Blot analysis for PARP-1 protein expression in the clones analyzed in (C). (**E**) Analysis of AID activity in DT40UNG^−/−^PARP-1^−/−^ and the respective parental and heterozygous cells. Data are representative of more than two independent experiments, statistical significance was determined using the two-sided Student's *t*-test. (**F**) Sequence analysis of the λ light chain locus in DT40UNG^−/−^cells and in three clones of DT40UNG^−/−^PARP-1^−/−^ cells shown in (F). Significance analysis: two-sided Fisher's exact test. Error bars show the standard deviation.

To analyze whether this occurs in the physiological context in B cells, and affects uracil generation by AID-mediated cytosine deamination rather than downstream repair pathways, we used DT40UNG^−/−^ (31) cells that allow for direct measurement of AID activity at the Ig locus via surface Ig (sIg) loss (Supplementary Figure S2A). Intriguingly, inhibition of PARP activity with two chemically different nicotinamide mimetics (TiqA and NU1025) led to a sharp increase of sIg loss in this cell system (Figure 3A), implying that PARP-1 activity restricts AID activity at the Ig locus.

To avoid effects due to differential selectivity of PARP inhibitors (40) and corroborate this finding by analyses in a defined genetic system, we decided to inactivate PARP-1 by gene targeting in DT40UNG^−/−^ cells. Using a targeting strategy resembling a previously successful one (Figure 3B), (41), we achieved PARP-1 locus inactivation (Figure 3C) and loss of PARP-1 protein (Figure 3D) in several independent clones. Analysis of sIg loss in selected clones of adequate genotype once again revealed increased AID activity at the Ig locus upon PARP-1 inactivation (Figure 3E), which was confirmed by sequencing (Figure 3F). We conclude that PARP-1 activity restricts AID activity at the Ig variable region, defining PARP-1 as the first factor capable of limiting AID activity at its physiological site of action. Apparently, this occurs by recruiting AID at DNA damage sites via PARylation, i.e. via a mechanism usually enabling DNA repair factor recruitment.

### PARP-1 activity restricts overall SHM in vitro

**Figure 4.**
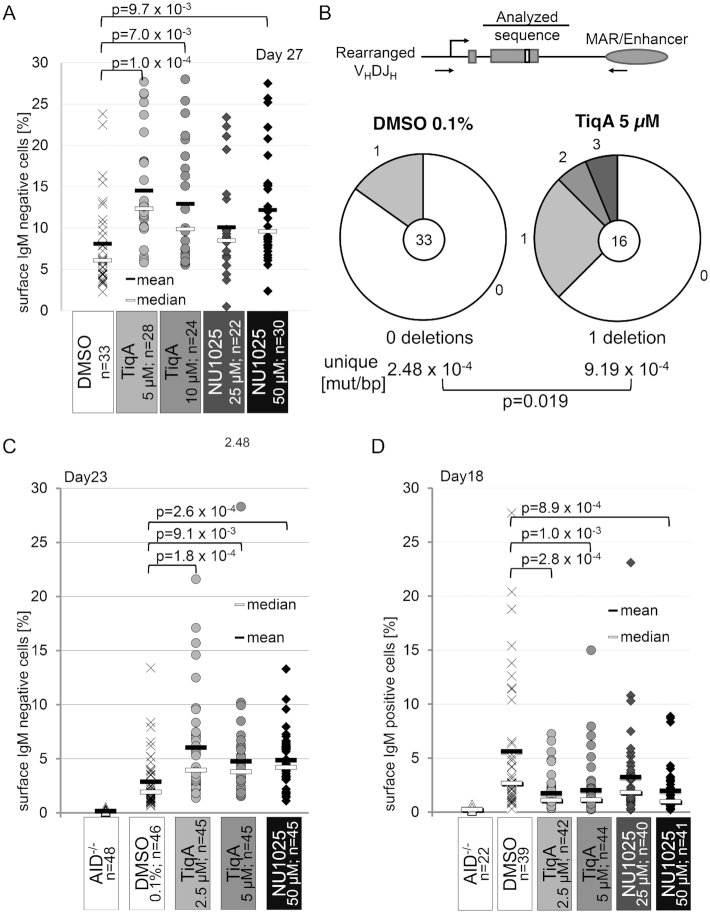
PARP inhibition leads to increased hypermutation *in vitro*. (**A**) Somatic hypermutation-mediated sIgM loss in RAMOS cells upon PARP-1 inhibition. Each dot indicates %sIgM loss in one single cell clone (see Supplementary Figure S2B for experimental system). One representative out of 3 experiments is shown. Significance analysis: two-sided Student's *t*-test. (**B**) Sequence analysis of the IgH variable region in representative RAMOS clones (from A) without and with PARP inhibition with 5 mM TiqA at day 42. Centers of the pie charts show numbers of analyzed sequences, relative amounts of sequences with the indicated number of mutations are given. Mutation frequencies are compared by a Fisher's exact test. (**C**) Effects of PARP inhibition on somatic hypermutation in DT40ΨV^−^ cells. Each dot indicates %IgM loss in one single cell clone (see Supplementary Figure S2C for experimental system). Data show one of two independent experiments. Statistically significant differences are marked with brackets, and P-values are given (two-sided Student's *t*-test). (**D**) Effect of PARP inhibition on Ig gene conversion in DT40Cre1 cells. Each dot indicates %IgM gain in one single cell clone (see Supplementary Figure S2D for experimental system). One representative out of two experiments shown. Significance analysis: two-sided Student's *t*-test.

We next asked how this PARP-1-mediated AID regulation would impact on the different processes of Ig diversification. In the hypermutating cell line RAMOS, inhibition of PARP activity led to an increase in sIg loss (Figure 4A), indicative of increased SHM (Figure 4B and Supplementary Figure S2B). Likewise, PARP inhibition led to increased sIg loss in hypermutating chicken DT40ψV^−^ cells (Figure 4C and Supplementary Figure S2C). Conversely, sIg gain in DT40Cre1 indicative of Ig gene conversion was decreased (Figure 4D and Supplementary Figure S2D), consistent with a previous study showing a crucial role of PARP-1 in homologous recombination, on which Ig gene conversion is based (42). Finally, CSR was moderately, if at all, increased by PARP inhibition in the CH12F3 cell line (Supplementary Figure S3A and B), variably affected by PARP inhibition in primary B cells (Supplementary Figure S3C and D) and even slightly decreased in B cells from PARP-1^−/−^ as compared to control mice (Supplementary Figure S3E). We conclude that inhibition of AID activity by PARP-1 concurs with inhibition of Ig variable region SHM in chicken and human cells, while effects of PARP-1 on DNA repair apparently overshadow its effects on AID in case of Ig gene conversion. For presently unknown reasons, CSR appears hardly affected by PARP-1-mediated AID inhibition.

### PARP-1 affects SHM *in vivo*

**Figure 5.**
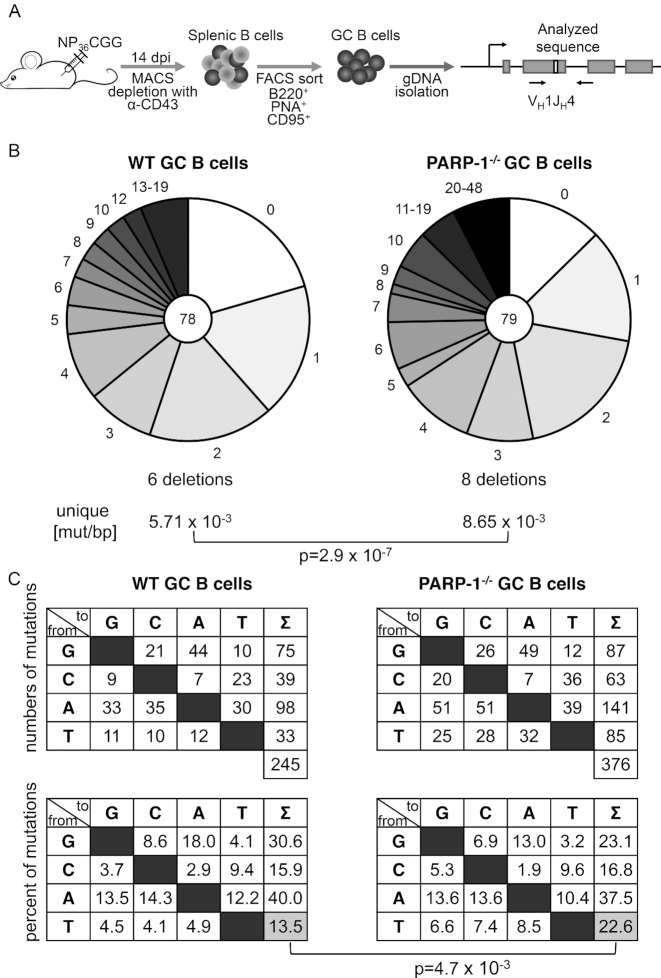
PARP-1 restricts somatic hypermutation *in vivo*. (**A**) Experimental setup to analyze somatic hypermutation in germinal center (GC) B cells of mice. (**B**) Sequence analysis of SHM in the JH_4_ intron in three pairs of WT and PARP-1^−/−^ mice. Numbers in the pie charts indicate numbers of analyzed sequences; relative amounts of sequences with the indicated number of mutations are given. Mutation frequencies are compared by a two-sided Fisher's exact test. (**C**) Mutation pattern (top: absolute, bottom: relative) of unique mutations in WT and PARP-1^−/−^ mice for the analysis shown in (B). Significance analysis: two-sided Fisher's exact test.

Finally, we wished to know whether the effects of PARP-1 on AID and SHM may also be detected in the context of a full germinal center reaction *in vivo*, i.e. under the conditions of iterative mutation and selection. For this, WT and PARP-1^−/−^ 129 mice were immunized with the model antigen NP-CGG, and germinal center B cells were sorted at day 14 after immunization (Figure 5A). Amplification and sequencing of the JH_4_ intron downstream of the V186.2 gene rearrangement used in the NP response revealed that overall hypermutation activity was indeed significantly enhanced in PARP-1^−/−^ mice (Figure 5B). Interestingly, analysis of the mutation pattern (Figure 5C) revealed significant changes as well, the most striking one being a reduction of the strand bias in A:T mutagenesis (i.e. an increase in relative mutations at T in PARP-1^-/-^ animals). We thus conclude that PARP-1 regulates AID and DNA repair during SHM at the Ig locus *in vitro* as well as in the *in vivo* situation.

## DISCUSSION

In the present study, we show that PARP-1 is a novel restriction factor of AID activity and SHM at the variable region of Ig genes (Supplementary Figure S3F). PARP-1 associates with AID in a DNA damage-dependent manner, leading to AID recruitment at DNA damage sites via AID-PAR association. This mechanism apparently serves to restrict the activity of AID at its site of action, i.e. directly at the Ig locus. Concomitantly, overall SHM is decreased by PARP-1 in chicken and human cell lines as well as in mouse germinal center B cells. Analysis of the mutation pattern reveals that PARP-1 also affects the repair of AID-induced lesions, since in addition to its known effect on homologous recombination repair (42) it also influences the mechanism underlying the strand bias of A:T mutagenesis, which is mostly elusive to date.

Our study was motivated by previous findings in our lab that implicated PARP-1 in the regulation of AID in case of exogenous introduction of DNA damage (20), which led to accumulation and stabilization of AID in the nucleus dependent on PARP-1 activity. Our present results shed more light on the mechanism of these previous findings. Apparently, the nuclear sequestration of AID is at least in part due to its direct recruitment to DNA damage sites by PARP-1-mediated PARylation. This mechanism resembles the recruitment of repair factors by PARP-1 to sites of DNA damage, but instead of activating AID function, it appears to serve as a blocking mechanism. One may thus presume that PARP-1 binding to a damaged gene locus, which then locally triggers AID recruitment by PARP-1, serves to protect this locus from further harm—a first example of a local negative feedback loop controlling AID activity. Intriguingly, during this time, AID is apparently not subject to nuclear degradation but rather stabilized, and might thus be released in its functional form once the damage, i.e. the strand break that immobilizes PARP-1 at the locus, is repaired. Another potential mechanism of AID release would be the action of the PARP counterplayer PARG that degrades the PAR chains (43). Such a cycle of AID-induced damage introduction, AID binding by PARP-1, and subsequent release by repair or PARG activity for resumed AID function is certainly highly intriguing and deserves further study, in particular relative to the described positive feedback cycle of DNA break-mediated amplification of AID activity via its phosphorylation at switch regions (44).

We have investigated the functional consequences of local AID inactivation via PARP-1 in most of the established model systems for the study of Ig diversification in vertebrates. It is important to note that these analyses were undertaken with the notion in mind that some previous studies on these issues were published, but did not in all cases yield consistent results. We will therefore address each mechanism of Ig diversification separately here.

In case of CSR, two previous studies have shown increased switching in cell lines upon PARP inhibition (45,46), but only one of these (46) could confirm the effect in primary mouse B cells, while the other one showed no effect there. A third study did describe altered class switching in mouse B cells lacking PARP-1 (47). While reasons for these conflicting data on the role of PARP-1 in class switch recombination are unclear, in our hands, effects of PARP-1 inhibition on switching were moderate at best, and variable. Based on the data available at this point, we consider it safe to say that PARP-1 does not substantially affect CSR efficiency as such. This is also consistent with the notion that while overall switching is similar in WT, PARP-1^−/−^ and PARP-2^−/−^ mice, different types of genetic alterations occurred in the two knockouts, implying that PARP-1 and 2 are active during the process but rather affect DNA repair than AID activity (46). Moreover, PARP-3 may provide an alternative control mechanism of CSR (48,49). Escape of AID from PARP-1 mediated restriction at switch regions (coupled to a positive break induced feedback loop (44)) may explain the high accumulation of strand breaks actually needed for double strand break formation and recombination to occur. In notable contrast, DNA breaks are not a required intermediate for the G:C biased phase of SHM, and only transiently affecting a single strand for the A:T mutator, giving meaning to two counterdirectional modes of DNA break-induced AID regulation during SHM and CSR. While underlying mechanisms are speculative at present, the relative efficiency of PAR chain competition with AID–DNA binding only via the substrate channel (as in the variable region) versus AID binding to structured/G4 DNA via substrate channel and assistant patch (as in switch regions (39)) should be investigated by adequate biochemical approaches.

In case of Ig gene conversion, our findings of reduced activity upon PARP-1 inactivation are fully consistent with a previous study showing a collapse of Ig gene conversion and activation of SHM upon PARP-1 inactivation in DT40 cells (42). Here, any potential effect of PARP-1 on AID function is likely fully masked by its impact on homologous recombination (41), i.e. DNA repair. Detection of PARP-1 effects on AID during both Ig gene conversion and CSR may thus be a challenging endeavor, requiring more sophisticated methods than applied here.

Concerning the role of PARP-1 in SHM, one early study claimed that PARP-1 is not required for the process (50), a statement actually true in its strict sense, We now show, though, that PARP-1 is an important negative regulator of SHM. In a setting where downstream repair events are blocked (DT40UNG^−/−^), the absence of PARP-1 leads to a profound increase in locus-specific AID activity. This finding also indicates that UNG-mediated generation of strand breaks may not be strictly required for recruitment of PARP-1 to the Ig locus. In all three investigated systems featuring the downstream repair pathways of SHM (human RAMOS, chicken DT40ψV−, mouse germinal center B cells), absence or inhibition of PARP-1 leads to an increased overall hypermutation. This may be due to effects on AID, DNA repair or both, and differentiation between these options is tricky when measuring the overall process. From a pragmatic point of view, we interpret an overall increase in SHM activity as an increase in AID activity, while changes in pattern would indicate changes in individual downstream repair pathways. Concerning the latter, we clearly detect an interesting pattern change in PARP-1^−/−^ mice: the strand bias of A:T mutagenesis, which appears to be quite pronounced in 129 mice, is alleviated. The mechanism generating this strand bias is elusive to date, even though several explanations have been put forth (51–53). Our identification of one unexpected responsible enzyme—PARP-1—may bring new air to its mechanistic investigation. Irrespective of this, we can safely conclude that PARP-1 regulates AID activity as well as DNA repair during Ig diversification.

In sum, we show for the first time that PARP-1 has a previously unanticipated role during SHM as a major restriction factor for AID function. Factors blocking aberrant activity of AID at its site of action have not previously been identified, and our findings may thus help to understand other pathways of AID/SHM regulation that are locus-specific. Importantly, both AID activity as well as error-prone lesion resolution are largely, but not fully, restricted to Ig gene loci, and the mechanism(s) responsible are barely understood to date. PARP-1 now presents as an enzyme with the potential to regulate both sides of the coin, and thus deserves much more rigorous investigation in the context of SHM than previously anticipated. Emphasizing this notion, locus perturbations leading to dominant negative PARP-1 proteins or decreased PARP-1 expression have been detected in Diffuse large B cell lymphoma (54), a disease characterized by aberrant somatic hypermutation (55), but nonetheless PARP inhibitor combination therapy is employed in the treatment of human B cell malignancies (56,57). On the basis of our findings, we advise caution with such approaches to prevent disease progression by deregulation of the dedicated mutator AID.

## Supplementary Material

gkz466_Supplemental_Files

## References

[1] Hoeijmakers J.H. Genome maintenance mechanisms for preventing cancer. Nature. 2001; 411:366–374. 11357144 10.1038/35077232

[2] Lombard D.B., Chua K.F., Mostoslavsky R., Franco S., Gostissa M., Alt F.W. DNA repair, genome stability, and aging. Cell. 2005; 120:497–512. 15734682 10.1016/j.cell.2005.01.028

[3] Swindall A.F., Stanley J.A., Yang E.S. PARP-1: friend or foe of DNA damage and repair in tumorigenesis. Cancers. 2013; 5:943–958. 24202328 10.3390/cancers5030943 PMC3795373

[4] Bürkle A., Virag L. Poly(ADP-ribose): PARadigms and PARadoxes. Mol. Aspects Med. 2013; 34:1046–1065. 23290998 10.1016/j.mam.2012.12.010

[5] Bürkle A. Physiology and pathophysiology of poly(ADP-ribosyl)ation. BioEssays. 2001; 23:795–806. 11536292 10.1002/bies.1115

[6] Krietsch J., Rouleau M., Pic E., Ethier C., Dawson T.M., Dawson V.L., Masson J.Y., Poirier G.G., Gagne J.P. Reprogramming cellular events by poly(ADP-ribose)-binding proteins. Mol. Aspects Med. 2013; 34:1066–1087. 23268355 10.1016/j.mam.2012.12.005 PMC3812366

[7] Jungnickel B. False moves for survival: error-prone DNA repair in adaptive immunity. Cell Cycle. 2006; 5:2856–2861. 17172871 10.4161/cc.5.24.3564

[8] Fugmann S.D., Schatz D.G. Immunology. One AID to unite them all. Science. 2002; 295:1244–1245. 11847327 10.1126/science.1070023

[9] Alt F.W., Zhang Y., Meng F.L., Guo C., Schwer B. Mechanisms of programmed DNA lesions and genomic instability in the immune system. Cell. 2013; 152:417–429. 23374339 10.1016/j.cell.2013.01.007 PMC4382911

[10] Arakawa H., Buerstedde J.M. Immunoglobulin gene conversion: insights from bursal B cells and the DT40 cell line. Dev Dyn. 2004; 229:458–464. 14991701 10.1002/dvdy.10495

[11] Chaudhuri J., Alt F.W. Class-switch recombination: interplay of transcription, DNA deamination and DNA repair. Nat. Rev. Immunol. 2004; 4:541–552. 15229473 10.1038/nri1395

[12] Di Noia J., Neuberger M.S. Altering the pathway of immunoglobulin hypermutation by inhibiting uracil-DNA glycosylase. Nature. 2002; 419:43–48. 12214226 10.1038/nature00981

[13] Di Noia J.M., Neuberger M.S. Molecular mechanisms of antibody somatic hypermutation. Annu. Rev. Biochem. 2007; 76:1–22. 17328676 10.1146/annurev.biochem.76.061705.090740

[14] Peña-Diaz J., Bregenhorn S., Ghodgaonkar M., Follonier C., Artola-Borán M., Castor D., Lopes M., Sartori Alessandro A., Jiricny J. Noncanonical mismatch repair as a source of genomic instability in human cells. Mol. Cell. 2012; 47:669–680. 22864113 10.1016/j.molcel.2012.07.006

[15] De Silva N.S., Klein U. Dynamics of B cells in germinal centres. Nat. Rev. Immunol. 2015; 15:137–148. 25656706 10.1038/nri3804 PMC4399774

[16] Keim C., Kazadi D., Rothschild G., Basu U. Regulation of AID, the B-cell genome mutator. Genes Dev. 2013; 27:1–17. 23307864 10.1101/gad.200014.112 PMC3553278

[17] Orthwein A., Di Noia J.M. Activation induced deaminase: how much and where. Semin. Immunol. 2012; 24:246–254. 22687198 10.1016/j.smim.2012.05.001

[18] Stavnezer J. Complex regulation and function of activation-induced cytidine deaminase. Trends Immunol. 2011; 32:194–201. 21493144 10.1016/j.it.2011.03.003 PMC3090464

[19] Zan H., Casali P. Regulation of Aicda expression and AID activity. Autoimmunity. 2013; 46:83–101. 23181381 10.3109/08916934.2012.749244 PMC3762583

[20] Tepper S., Jeschke J., Bottcher K., Schmidt A., Davari K., Muller P., Kremmer E., Hemmerich P., Pfeil I., Jungnickel B. PARP activation promotes nuclear AID accumulation in lymphoma cells. Oncotarget. 2016; 7:13197–13208. 26921193 10.18632/oncotarget.7603 PMC4914351

[21] Greiner A., Tobollik S., Buettner M., Jungnickel B., Herrmann K., Kremmer E., Niedobitek G. Differential expression of activation-induced cytidine deaminase (AID) in nodular lymphocyte-predominant and classical Hodgkin lymphoma. J. Pathol. 2005; 205:541–547. 15732141 10.1002/path.1746

[22] Fischbach A., Krüger A., Hampp S., Assmann G., Rank L., Hufnagel M., Stöckl M.T., Fischer J.M.F., Veith S., Rossatti P. et al. The C-terminal domain of p53 orchestrates the interplay between non-covalent and covalent poly(ADP-ribosyl)ation of p53 by PARP1. Nucleic Acids Res. 2018; 46:804–822. 29216372 10.1093/nar/gkx1205 PMC5778597

[23] Coker H.A., Petersen-Mahrt S.K. The nuclear DNA deaminase AID functions distributively whereas cytoplasmic APOBEC3G has a processive mode of action. DNA Repair (Amst.). 2007; 6:235–243. 17161027 10.1016/j.dnarep.2006.10.001

[24] Rangam G., Schmitz K.-M., Cobb A.J.A., Petersen-Mahrt S.K. AID enzymatic activity is inversely proportional to the size of cytosine C5 orbital cloud. PLoS One. 2012; 7:e43279. 22916236 10.1371/journal.pone.0043279 PMC3423351

[25] Fahrer J., Kranaster R., Altmeyer M., Marx A., Bürkle A. Quantitative analysis of the binding affinity of poly(ADP-ribose) to specific binding proteins as a function of chain length. Nucleic Acids Res. 2007; 35:e143. 17991682 10.1093/nar/gkm944 PMC2175335

[26] Xie S., Mortusewicz O., Ma H.T., Herr P., Poon R.R., Helleday T., Qian C. Timeless interacts with PARP-1 to promote homologous recombination repair. Mol. Cell. 2015; 60:163–176. 26344098 10.1016/j.molcel.2015.07.031

[27] Sanchez-Molina S., Mortusewicz O., Bieber B., Auer S., Eckey M., Leonhardt H., Friedl A.A., Becker P.B. Role for hACF1 in the G2/M damage checkpoint. Nucleic Acids Res. 2011; 39:8445–8456. 21745822 10.1093/nar/gkr435 PMC3201854

[28] Herce H.D., Deng W., Helma J., Leonhardt H., Cardoso M.C. Visualization and targeted disruption of protein interactions in living cells. Nat. Commun. 2013; 4:2660. 24154492 10.1038/ncomms3660 PMC3826628

[29] Shanbhag N.M., Rafalska-Metcalf I.U., Balane-Bolivar C., Janicki S.M., Greenberg R.A. ATM-dependent chromatin changes silence transcription in cis to DNA double-strand breaks. Cell. 2010; 141:970–981. 20550933 10.1016/j.cell.2010.04.038 PMC2920610

[30] Arakawa H., Saribasak H., Buerstedde J.M. Activation-induced cytidine deaminase initiates immunoglobulin gene conversion and hypermutation by a common intermediate. PLoS Biol. 2004; 2:E179. 15252444 10.1371/journal.pbio.0020179 PMC449846

[31] Saribasak H., Saribasak N.N., Ipek F.M., Ellwart J.W., Arakawa H., Buerstedde J.M. Uracil DNA glycosylase disruption blocks Ig gene conversion and induces transition mutations. J. Immunol. 2006; 176:365–371. 16365429 10.4049/jimmunol.176.1.365

[32] Arakawa H., Lodygin D., Buerstedde J.M. Mutant loxP vectors for selectable marker recycle and conditional knock-outs. BMC Biotech. 2001; 1:7. 10.1186/1472-6750-1-7 PMC57747 11591226

[33] Maccarthy T., Roa S., Scharff M.D., Bergman A. SHMTool: a webserver for comparative analysis of somatic hypermutation datasets. DNA Repair (Amst.). 2009; 8:137–141. 18952008 10.1016/j.dnarep.2008.09.006 PMC2659805

[34] Scheller H., Tobollik S., Kutzera A., Eder M., Unterlehberg J., Pfeil I., Jungnickel B. c-Myc overexpression promotes a germinal center-like program in Burkitt's lymphoma. Oncogene. 2010; 29:888–897. 19881537 10.1038/onc.2009.377

[35] Ogata N., Ueda K., Kawaichi M., Hayaishi O. Poly(ADP-ribose) synthetase, a main acceptor of poly(ADP-ribose) in isolated nuclei. J. Biol. Chem. 1981; 256:4135–4137. 6260786

[36] Peralta-Leal A., Rodriguez-Vargas J.M., Aguilar-Quesada R., Rodriguez M.I., Linares J.L., de Almodovar M.R., Oliver F.J. PARP inhibitors: new partners in the therapy of cancer and inflammatory diseases. Free Radic. Biol. Med. 2009; 47:13–26. 19362586 10.1016/j.freeradbiomed.2009.04.008

[37] Curtin N.J., Szabo C. Therapeutic applications of PARP inhibitors: anticancer therapy and beyond. Mol. Aspects Med. 2013; 34:1217–1256. 23370117 10.1016/j.mam.2013.01.006 PMC3657315

[38] Mortusewicz O., Ame J.C., Schreiber V., Leonhardt H. Feedback-regulated poly(ADP-ribosyl)ation by PARP-1 is required for rapid response to DNA damage in living cells. Nucleic Acids Res. 2007; 35:7665–7675. 17982172 10.1093/nar/gkm933 PMC2190722

[39] Qiao Q., Wang L., Meng F.L., Hwang J.K., Alt F.W., Wu H. AID recognizes structured DNA for class switch recombination. Mol. Cell. 2017; 67:361–373. 28757211 10.1016/j.molcel.2017.06.034 PMC5771415

[40] Wahlberg E., Karlberg T., Kouznetsova E., Markova N., Macchiarulo A., Thorsell A.-G., Pol E., Frostell A., Ekblad T., Oncu D. et al. Family-wide chemical profiling and structural analysis of PARP and tankyrase inhibitors. Nat. Biotechnol. 2012; 30:283–288. 22343925 10.1038/nbt.2121

[41] Hochegger H., Dejsuphong D., Fukushima T., Morrison C., Sonoda E., Schreiber V., Zhao G.Y., Saberi A., Masutani M., Adachi N. et al. Parp-1 protects homologous recombination from interference by Ku and Ligase IV in vertebrate cells. EMBO J. 2006; 25:1305–1314. 16498404 10.1038/sj.emboj.7601015 PMC1422167

[42] Paddock M.N., Buelow B.D., Takeda S., Scharenberg A.M. The BRCT domain of PARP-1 is required for immunoglobulin gene conversion. PLoS Biol. 2010; 8:e1000428. 20652015 10.1371/journal.pbio.1000428 PMC2907289

[43] Tanuma S., Sato A., Oyama T., Yoshimori A., Abe H., Uchiumi F. New Insights into the Roles of NAD+-Poly(ADP-ribose) Metabolism and Poly(ADP-ribose) Glycohydrolase. Curr. Protein Peptide Sci. 2016; 17:668–682. 27817743 10.2174/1389203717666160419150014

[44] Vuong B.Q., Herrick-Reynolds K., Vaidyanathan B., Pucella J.N., Ucher A.J., Donghia N.M., Gu X., Nicolas L., Nowak U., Rahman N. et al. A DNA break- and phosphorylation-dependent positive feedback loop promotes immunoglobulin class-switch recombination. Nat. Immunol. 2013; 14:1183–1189. 24097111 10.1038/ni.2732 PMC4005274

[45] Shockett P., Stavnezer J. Inhibitors of poly(ADP-ribose) polymerase increase antibody class switching. J. Immunol. 1993; 151:6962–6976. 8258703

[46] Robert I., Dantzer F., Reina-San-Martin B. Parp1 facilitates alternative NHEJ, whereas Parp2 suppresses IgH/c-myc translocations during immunoglobulin class switch recombination. J. Exp. Med. 2009; 206:1047–1056. 19364882 10.1084/jem.20082468 PMC2715026

[47] Ambrose H.E., Willimott S., Beswick R.W., Dantzer F., de Murcia J.M., Yelamos J., Wagner S.D. Poly(ADP-ribose) polymerase-1 (Parp-1)-deficient mice demonstrate abnormal antibody responses. Immunology. 2009; 127:178–186. 18778284 10.1111/j.1365-2567.2008.02921.x PMC2691783

[48] Rulten S.L., Fisher A.E., Robert I., Zuma M.C., Rouleau M., Ju L., Poirier G., Reina-San-Martin B., Caldecott K.W. PARP-3 and APLF function together to accelerate nonhomologous end-joining. Mol. Cell. 2011; 41:33–45. 21211721 10.1016/j.molcel.2010.12.006

[49] Robert I., Gaudot L., Rogier M., Heyer V., Noll A., Dantzer F., Reina-San-Martin B. Parp3 negatively regulates immunoglobulin class switch recombination. PLos Genet. 2015; 11:e1005240. 26000965 10.1371/journal.pgen.1005240 PMC4441492

[50] Jacobs H., Fukita Y., van der Horst G.T., de Boer J., Weeda G., Essers J., de Wind N., Engelward B.P., Samson L., Verbeek S. et al. Hypermutation of immunoglobulin genes in memory B cells of DNA repair-deficient mice. J. Exp. Med. 1998; 187:1735–1743. 9607915 10.1084/jem.187.11.1735 PMC2212309

[51] Zivojnovic M., Delbos F., Girelli Zubani G., Jule A., Alcais A., Weill J.C., Storck S. Somatic hypermutation at A/T-rich oligonucleotide substrates shows different strand polarities in Ung-deficient or -proficient backgrounds. Mol. Cell Biol. 2014; 34:2176–2187. 24710273 10.1128/MCB.01452-13 PMC4054293

[52] Kano C., Wang J.Y. High levels of AID cause strand bias of mutations at A versus T in Burkitt's lymphoma cells. Mol. Immunol. 2013; 54:397–402. 23399385 10.1016/j.molimm.2013.01.005

[53] Mayorov V.I., Rogozin I.B., Adkison L.R., Gearhart P.J. DNA polymerase eta contributes to strand bias of mutations of A versus T in immunoglobulin genes. J. Immunol. 2005; 174:7781–7786. 15944281 10.4049/jimmunol.174.12.7781

[54] de Miranda N.F., Peng R., Georgiou K., Wu C., Falk Sorqvist E., Berglund M., Chen L., Gao Z., Lagerstedt K., Lisboa S. et al. DNA repair genes are selectively mutated in diffuse large B cell lymphomas. J. Exp. Med. 2013; 210:1729–1742. 23960188 10.1084/jem.20122842 PMC3754869

[55] Pasqualucci L., Neumeister P., Goossens T., Nanjangud G., Chaganti R.S., Kuppers R., Dalla-Favera R. Hypermutation of multiple proto-oncogenes in B-cell diffuse large-cell lymphomas. Nature. 2001; 412:341–346. 11460166 10.1038/35085588

[56] Soumerai J.D., Zelenetz A.D., Moskowitz C.H., Palomba M.L., Hamlin P.A. Jr, Noy A., Straus D.J., Moskowitz A.J., Younes A., Matasar M.J. et al. The PARP inhibitor veliparib can be safely added to bendamustine and rituximab and has preliminary evidence of activity in B-Cell Lymphoma. Clin. Cancer Res. 2017; 23:4119–4126. 28314788 10.1158/1078-0432.CCR-16-3068 PMC5541854

[57] Kummar S., Ji J., Morgan R., Lenz H.J., Puhalla S.L., Belani C.P., Gandara D.R., Allen D., Kiesel B., Beumer J.H. et al. A phase I study of veliparib in combination with metronomic cyclophosphamide in adults with refractory solid tumors and lymphomas. Clin. Cancer Res. 2012; 18:1726–1734. 22307137 10.1158/1078-0432.CCR-11-2821 PMC3306481

