# Supplementary material for: Restriction of AID activity and somatic hypermutation by PARP-1
Source: Nucleic Acids Res. 2019 May 25;47(14):7418–29. doi: 10.1093/nar/gkz466 (PMC6698665; doi:10.1093/nar/gkz466)
Supplement: gkz466_Supplemental_Files [file gkz466_supplemental_files.pdf]

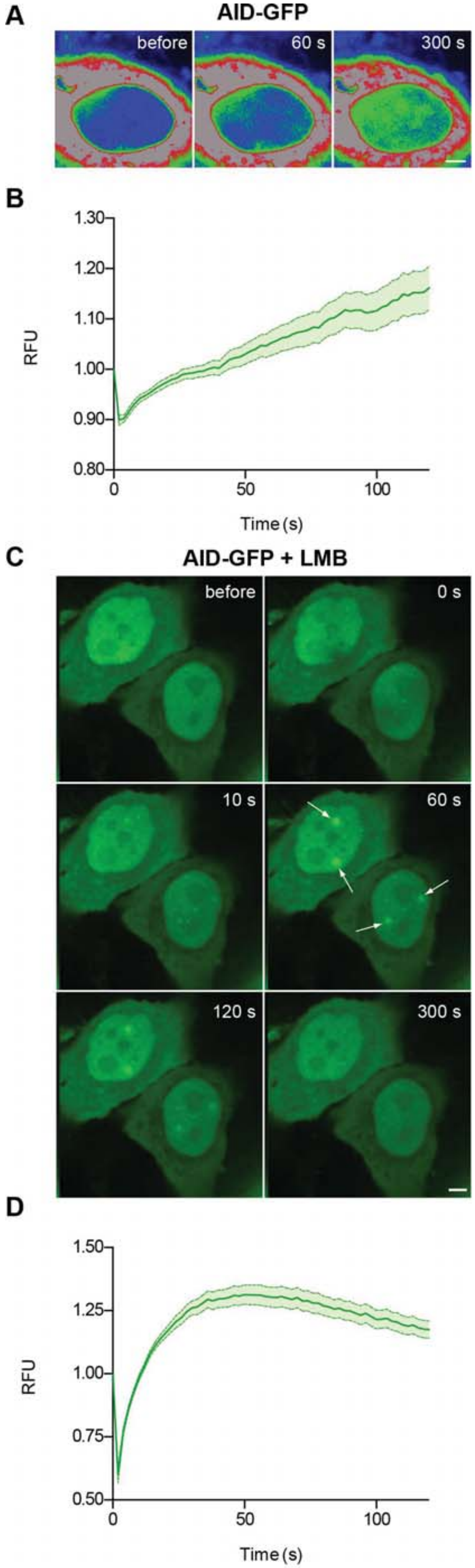

Figure S1: Nuclear accumulation and recruitment of AID-GFP to DNA damage sites

A) Representative false-color images of AID-GFP accumulation in the nucleus after laser microirradiation in HelaKyoto cells. **B)** Quantification of AID accumulation in the nucleus. **C)** Representative images of AID-GFP recruitment to laser-induced DNA damage sites in the presence of the nuclear export inhibitor LMB (20 ng/ml, 6 h pre-treatment). **D)** Quantification of the results shown in C. Data of 44 (B) and 26 (D) cells from two (D) or three (B) independent experiments are shown as mean ± SEM. Microirradiation was performed using a spinning disk microscope. Scale bar, 5 µm. RFU = Relative fluorescence units.

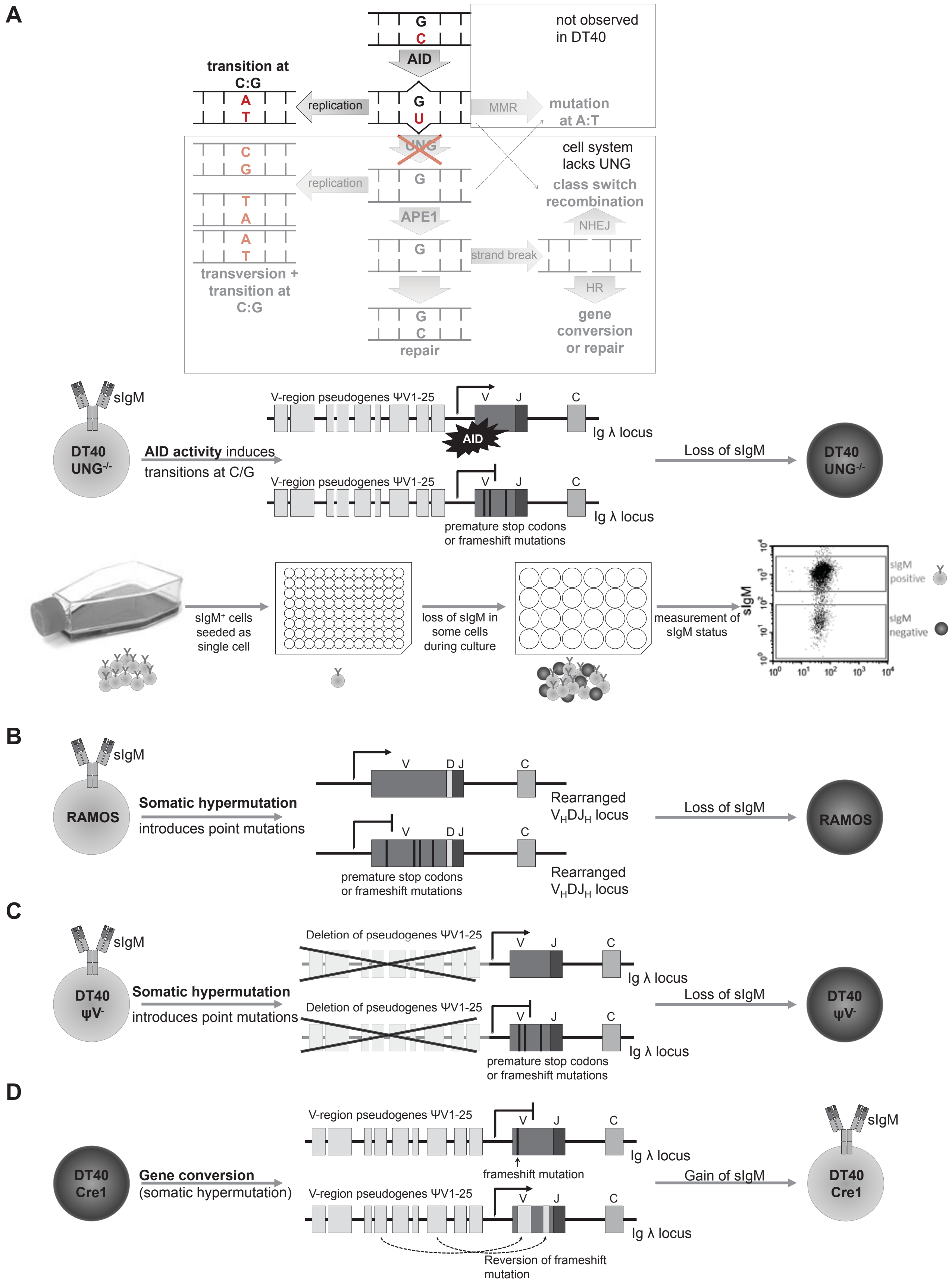

Figure S2: Systems for AID activity in DT40 and Ig diversification in RAMOS or DT40

A) System for the analysis of AID activity in DT40UNG$^{-/-}$ cells. Inactivation of UNG blocks Ig gene conversion and only allows transition mutations in Ig genes (due to the lack of an A:T mutator in DT40 cells), which are indicative of AID activity. Some of these mutations lead to surface Ig loss, which can be quantified by FACS measurements of multiple individual single cell clones that were initially sIg^{+} and lose sIg upon culture. **B)** System for measurement of somatic hypermutation in RAMOS. Mutations in Ig genes may lead to sIg loss that was quantified as described in A). **C)** System for measurement of hypermutation in DT40ΨV^{-} cells. Deletion of the pseudogenes required for Ig gene conversion leads to a switch to somatic hypermutation. **D)** System for measurement of Ig gene conversion in DT40Cre1 cells. Repair of a frameshift mutation in the original cells by AID-induced homologous recombination with upstream pseudogenes leads to sIg gain.

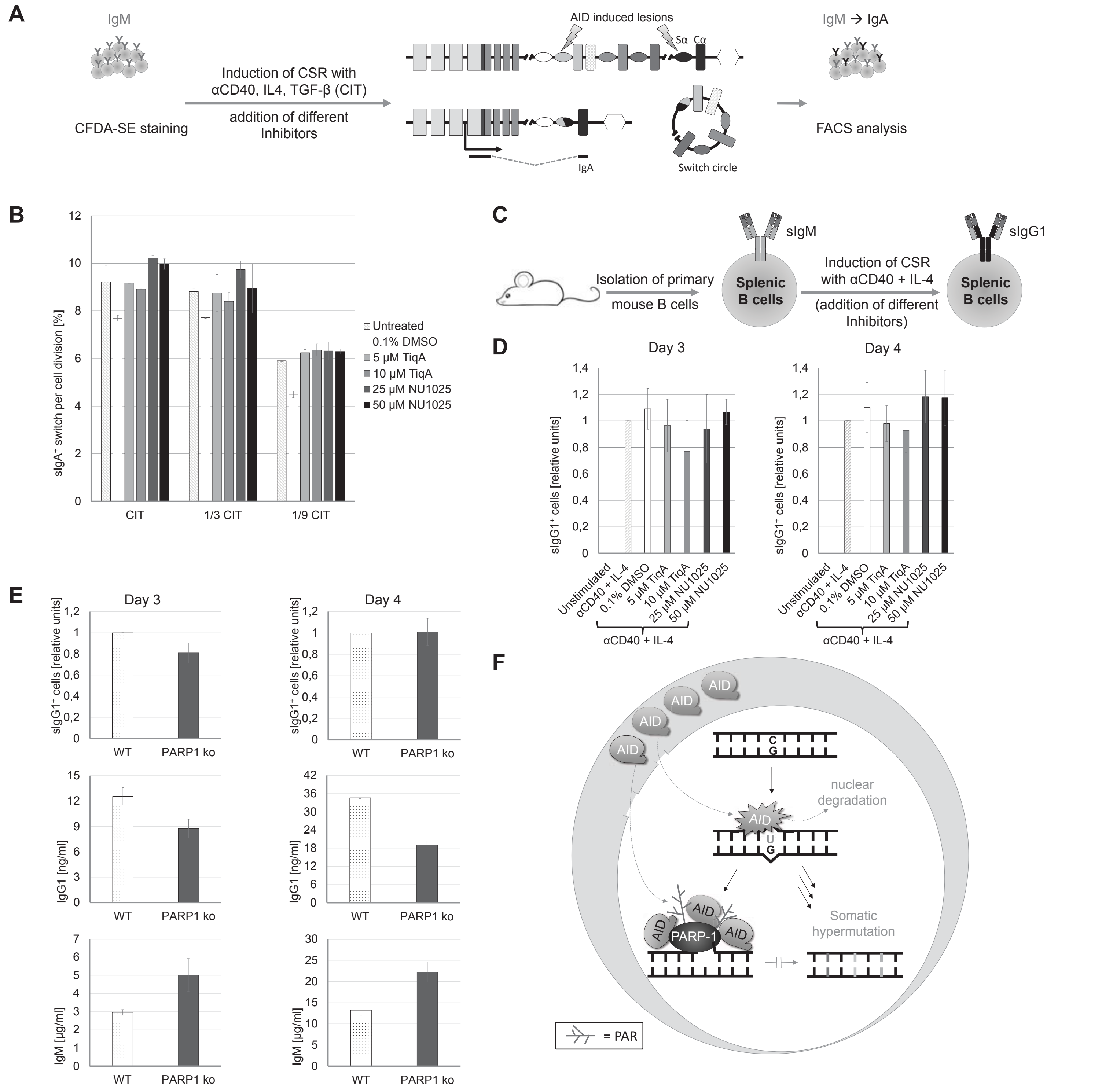

Figure S3: Effects of PARP inhibition on class switch recombination

A) System for analysis of class switch recombination in CH12F3 cells. Stimulation of the cells with αCD40, IL-4 and TGFβ (CIT) leads to class switching from IgM to IgA, which can be quantified by FACS. **B)** Switching per cell division (as measured by CFSE dilution) upon PARP inhibition in CH12F3 cells. **C)** System for analysis of class switch recombination in primary mouse B cells. Stimulation with αCD40 and IL-4 leads to class switching to IgG1 which is measured by FACS. **D)** Effects of PARP inhibition on CSR in primary mouse cells. CSR data of 3 independent experiments were normalized to samples treated with αCD40 and IL-4. **E)** Switch recombination efficiency in WT vs. PARP-1$^{-/-}$ mouse B cells, as well as IgG1 and IgM production, measured at day 3 and 4. Data for CSR are derived from 3 independent experiments and were normalized to switching induced in WT cells. **F)** Model for regulation of AIDs activity via PARP-1 and PARylation.
